# Defining the optimal cryoprotectant and concentration for cryopreservation of limbal stem cells

**DOI:** 10.1016/j.cryobiol.2018.07.008

**Published:** 2018-10

**Authors:** Charles Osei-Bempong, Ali E. Ghareeb, Majlinda Lako, Francisco C. Figueiredo, W. John Armitage

**Affiliations:** a Institute of Genetic Medicine, Newcastle University, International Centre for Life, Central Parkway, Newcastle upon Tyne, NE1 3BZ, UK; b Department of Ophthalmology, Royal Victoria Infirmary, Newcastle University, Newcastle upon Tyne, UK; c Division of Ophthalmology, University of Bristol, Bristol Eye Hospital, Lower Maudlin Street, Bristol BS1 2LX, UK

**Keywords:** Cryopreservation, Eye bank, Cornea, Corneal transplant, Limbal stem cells, Limbal stem cell deficiency, Dimethyl sulphoxide, Propylene glycol, Ethylene glycol, Cell tolerance

## Abstract

Limbal stem cell (LSC) deficiency causes progressive loss of vision but may be treated by transplant of autologous LSCs. Cryopreservation has the potential to indefinitely extend the lifespan of LSCs allowing re-transplant in case of graft failure. In this study, we aimed to identify the optimal cryoprotectant and cryoprotectant concentration for LSC cultures. Suspension cultures derived from cadaveric corneoscleral rims were cooled to 4 °C with Me_2_SO, propylene glycol or ethylene glycol at a concentration of 5%, 10% or 15%. Cell tolerance was measured in terms of membrane integrity, colony-forming efficiency and alamarBlue^®^ reduction. Increasing cryoprotectant concentration above 5% reduced membrane integrity, metabolism and colony-forming efficiency. Cryoprotectant choice did not significantly influence these characteristics. Cells demonstrating Side Population were maintained after cryopreservation with 5% propylene glycol in vapour phase liquid nitrogen for 1 week, indicating that cryopreservation of LSCs with relatively low cryoprotectant concentration (5%) has promise in low-temperature eye banking.

## Main article

The limbus is a fine ring of tissue which borders the cornea and is the site of corneal progenitors known as limbal stem cells [12]. Limbal stem cell deficiency is a rare cause of blindness in which there are too few healthy limbal stem cells to carry out tissue regeneration [10]. Total limbal stem cell deficiency has previously been successfully treated with transplantation of healthy limbal tissue [11,13]. In unilateral disease this has been an autologous transplant, with the limbal tissue being taken from the healthy eye. Although effective in improving vision in the diseased eye, this technique risked jeopardising the limbal stem cells in the healthy eye as significant amounts of limbal tissue are required for transplantation. To circumvent this risk, small limbal biopsies may be taken from the healthy limbus and cultured *in vitro* [11]. Two strategies are employed: one is to expand the limbal biopsies on amniotic membrane while the other is expansion in suspension culture [8]. Few cells are required to seed suspension cultures and the cells are transferred to a scaffold to facilitate epithelium layer formation. This represents an autologous transplant and is conducted under good manufacturing practice (GMP) conditions in several clinical facilities worldwide [5,8]. In the UK, few centres can offer both GMP culturing facilities and surgical expertise. Therefore, dissemination of cultured material will be required, both for research and clinical transplantation [8]. Furthermore, cryopreservation of limbal suspension cultures is beneficial as it obviates the need for further biopsies in the case of graft failure [5].

Cryopreservation has the potential to make *ex-vivo* expanded limbal epithelial cells readily available for transplant by extending the lifespan of limbal suspension cultures in eye banks indefinitely [2]. An extensive investigation of optimal cryopreservation conditions is essential to improve low-temperature banking of limbal stem cell suspension cultures.

Our aims were twofold: to identify optimal cryopreservation conditions from a group of candidate cryoprotectants employed at different concentrations and to look for the presence of the Side Population (SP) phenotype in cryopreserved limbal epithelial cultures. Stem cells show the ability to efflux Hoechst 33,342 (a DNA binding dye which binds preferentially at A-T rich regions) via the transmembrane protein ABCG2. Hoechst 33,342 is excited by UV light and emits in the blue range (395 nm excitation, 450 nm emission). This gives rise to the characteristic of SP which can be detected by flow cytometric analysis and which has been demonstrated in various adult cell cultures, including those derived from bone, lung, liver and skeletal muscle. The SP characteristic can be verified by Verapamil which blocks both ABCG1/2 transporters, and Fumitremorgin, an agent that specifically inhibits ABCG2 [4,16].

Human limbal epithelial cells were cultured from intact cadaveric corneoscleral rims, stored in organ culture in the NHS Blood and Transplant Eye Banks in Bristol and Manchester and supplied with consent for research. Cells in suspension were co-cultured with 3T3 mouse fibroblasts which facilitate the *ex-vivo* expansion of limbal stem cells [10].

Medium for the 3T3 mouse fibroblast feeders was composed of high glucose Dulbecco's modified Eagle's medium, 10% FCS and 1% penicillin/streptomycin [all from Invitrogen]. Limbal epithelial culture medium was as described in Ahmad et al. [1]. A carrier medium provided a vehicle for suspended cells to be bathed in along with cryoprotectant. Carrier medium consisted of Dulbecco's Modified Eagles Medium (66.75%), Ham's F12 (22.25%), FCS (10%) and penicillin/streptomycin (1%) [all Invitrogen, UK]. This carrier medium excluded additives which could precipitate out of solution at low temperatures.

Murine 3T3 fibroblasts were seeded at a density of 24,000 cells/cm^2^. Every third day the 3T3 medium was changed and the cells were passaged at the point of confluence. Inactivation was achieved by irradiation with 6Gy of X-ray at 120 kV, 4 mA for 7 min [Faxitron CP-160]. The 3T3 mouse fibroblasts were released from the cell culture plastic with 0.05% trypsin +0.53 mM ethylene diaminetetraacetic acid, re-suspended in 3T3 medium and plated at a density of 24,000 cells/cm^2^ per well of a 6 well plate [TPP]. All cultures were incubated at 37 °C, 5% CO_2_ with humidified air.

Limbal suspension cultures were obtained from the corneoscleral rims of 9 donors as described in Ahmad et al. [1] and co-cultured with 3T3 mouse fibroblasts at a density of 15,000 cell/cm^2^. The culture medium was changed on the third day and every second day subsequently.

The alamarBlue^®^ bioassay has been used to measure cytotoxicity and cell viability in a range of experimental systems over the past 50 years [14]. AlamarBlue^®^, also known as resazurin (blue and non-fluorescent), is reduced to resorufin (pink and highly fluorescent) in the presence of cellular metabolism by an oxidation reaction.

Before designing the alamarBlue^®^ experiments, we first tested the suitability of the assay in detecting overall changes in levels of cellular metabolism in limbal suspension cultures. Cells were released from culture wells as described and re-suspended in carrier medium. Cells were then plated at densities of 1000, 5000, 10,000 or 20,000 cells/cm^2^ for the alamarBlue^®^ suitability assay, and at a density of 5000 cells/cm^2^ for the cell tolerance assays. The suspension culture was seeded in 100 μl aliquots and incubated with 10 μl of alamarBlue^®^ [Bio-Rad] in a 96 well plate [Sigma]. A fully reduced sample was also included as a positive control to denote the upper limit of fluorescence. Fully reduced alamarBlue^®^ was made by autoclaving 100 μl of alamarBlue^®^ in 1 ml of carrier medium for 90 min (121 °C, 2192 mBar, Astell). For the alamarBlue^®^ suitability assay, incubation was for 6 or 12 h and the fluorescence was measured at 544 nm excitation, 585 nm emission [Fluoroskan, Ascent] at the end of incubation. In the cell tolerance experiments, all wells were incubated with alamarBlue^®^ for 12 h. Optical adhesive film [Applied Biosystems, UK] was used to reduce evaporation. Values were expressed as a percentage of the positive control. Irradiated limbal cells were exposed to 6Gy of X-ray radiation emitted at 120 kV, 4 mA for 7 min [Faxitron CP-160] before being incubated with alamarBlue^®^. Cells were killed by direct freezing to −80 °C without cryoprotectant. They were stored frozen overnight and rewarmed by sitting at room temperature the next day.

Trypan blue (0.4%) [Invitrogen] and a haemocytometer were used to determine the percentage of cells with intact membranes before and after each cryoprotectant tolerance experiment. A 10 μl aliquot was observed in a haemocytometer under phase microscopy. Membrane integrity is expressed as the number of cells with intact membranes as a percentage of the total number of cells.

The colony-forming efficiency assay (CFE) was performed as in Ahmad et al. [1].

In preparation for cooling, cultured epithelial cells were released from the 6 well plate (TTP) as described. The cell suspension was centrifuged at 1000 RPM, re-suspended in carrier medium, then divided into four aliquots, and a membrane integrity assay was performed on each sample as described. Each sample was then mixed with 10%, 20% or 30% (v/v) of the cryoprotectant in an equal volume of carrier medium at 4 °C to achieve final concentrations of 5%, 10% and 15%. The samples were then held at 4 °C in the chamber of a controlled rate freezer [Kryo 560–16, Planer] for 10 min. The 0% samples (positive controls) were not cooled. In total, the cells were exposed to the cryoprotectants for 10 min before serial dilution. At the end of the 10-min interval all samples were removed and diluted twice with equal volumes of carrier medium. The samples were centrifuged at 1000 rpm and the supernatant was removed. The resulting pellet was re-suspended in carrier medium. Ice nucleation was not employed for these experiments but was employed for the SP experiments (see next paragraph). As the cells and cryoprotectants were held at 4 °C for the duration of the experiment, no cooling rate was recorded. Next, the trypan blue assay was performed on each sample and colony-forming efficiencies were then calculated based on the number of membrane intact epithelial cells counted. The effect of cryoprotectant and cryoprotectant concentration was analysed by 2-way ANOVA.

For the SP experiments, limbal cultures suspended in 500 μl were made up to 1 ml with Icestart™ [Asymptote]. Propylene glycol (10%) at 4 °C was then added to give a final concentration of 5%. The samples were then transferred into 1.8 ml Cryovials [Nunc (Thermo Fisher)]. A Kryo 560–16 controlled rate freezer [Planer] was used to cool samples according to the following protocol: (I) 4 °C, held for 10 min (II) −2 °C/min to −5 °C (III) −1 °C/min to −40 °C (IV) −5 °C/min to −100 °C. On completion of the program, samples were stored in the vapour phase of liquid nitrogen dewars [Taylor-Wharton] for 7 days. Samples were rapidly thawed at 37 °C in a water bath.

Hank's solution was prepared with 500 mls HBSS [Sigma] containing 2% FCS [Invitrogen], and 5 ml of 5000 units of pen/strep [Gibco]. Cells were detached from culture wells as described and resuspended to 5 × 10^5^ cells/ml. Detached cells were split into three groups and either stained with Hoechst 33,342 dye alone, with Hoechst 33,342 and Verapamil (10 μM), or Hoechst 33,342 and Fumitremorgin (5 μM) for 45 min at 37 °C, 5% CO_2_. All treatments were rotated at 37 °C in 5% CO_2_ in a MACsMix [Mitenyi Biotec] for 30 min and cold PBS or Hank's solution was used to terminate reactions. To optimise the Hoechst concentration for limbal SP cells a range of Hoechst concentrations were tested (data not shown). All cell suspensions were pelleted at 2500 rpm for 5 min and the supernatant containing the dye was removed. Medium was used to resuspend the pelleted cell into a FACS tube, after straining with a 70 μm filter [BD Falcon, UK]. A BD LSRII flow cytometer was used, and data was analysed using BD FACSDiva software™ (BD Biosciences, US).

**Fig. 1:**
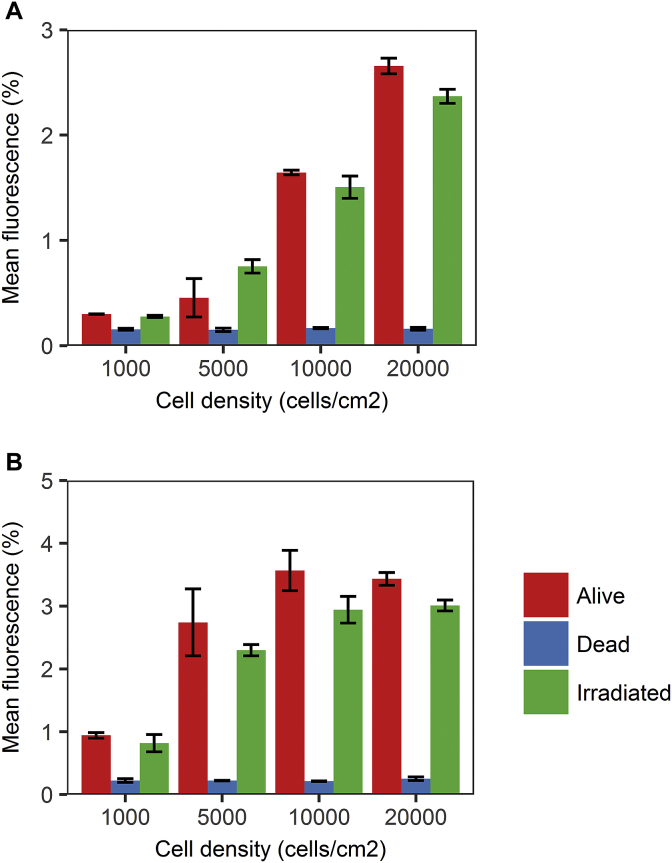
Bar graphs showing mean alamarBlue^®^ fluorescence for alive, irradiated and dead limbal suspension cultures at cell densities of 1000, 5000, 10,000 and 20,000 cells/cm^2^. **(A)** mean fluorescence ± SEM for a given cell density after 6 h of incubation with alamarBlue^®^ (n = 3). Mean fluorescence was calculated as a percentage of the fluorescence of the positive controls. (**B)** mean fluorescence ± SEM for a given cell density after 12 h of incubation with alamarBlue^®^ (n = 3). Mean fluorescence was calculated as a percentage of the fluorescence of the positive controls.

For cultures incubated with alamarBlue^®^ for 6 h, reduction of alamarBlue^®^ was proportion to cell density for both living and irradiated cultures (Fig. 1A). In cultures incubated for 12 h, alamarBlue^®^ reduction was proportional to cell density up to a density of 10,000 cells/cm^2^, after which it plateaued (Fig. 1B). This is most likely due to the inhibitory effects of high cell density on metabolic activity as resources become limited. Dead cells reduced alamarBlue^®^ at a low level regardless of cell density. From these results, we concluded that alamarBlue^®^ would form a suitable assay of metabolism for limbal suspension cultures at a density of 5000 cell/cm^2^ and 12 h of incubation.

The following cryoprotectants were selected as they are liquid at room temperature and are commonly used: Me_2_SO, propylene glycol, (1,3-propanediol; PD) and ethylene glycol (ethane-1,2-diol, EG) [6,7]. Glycerol was not tested because it induces a large degree of osmotic stress and cells require a long exposure time before cryopreservation [7].

**Fig. 2:**
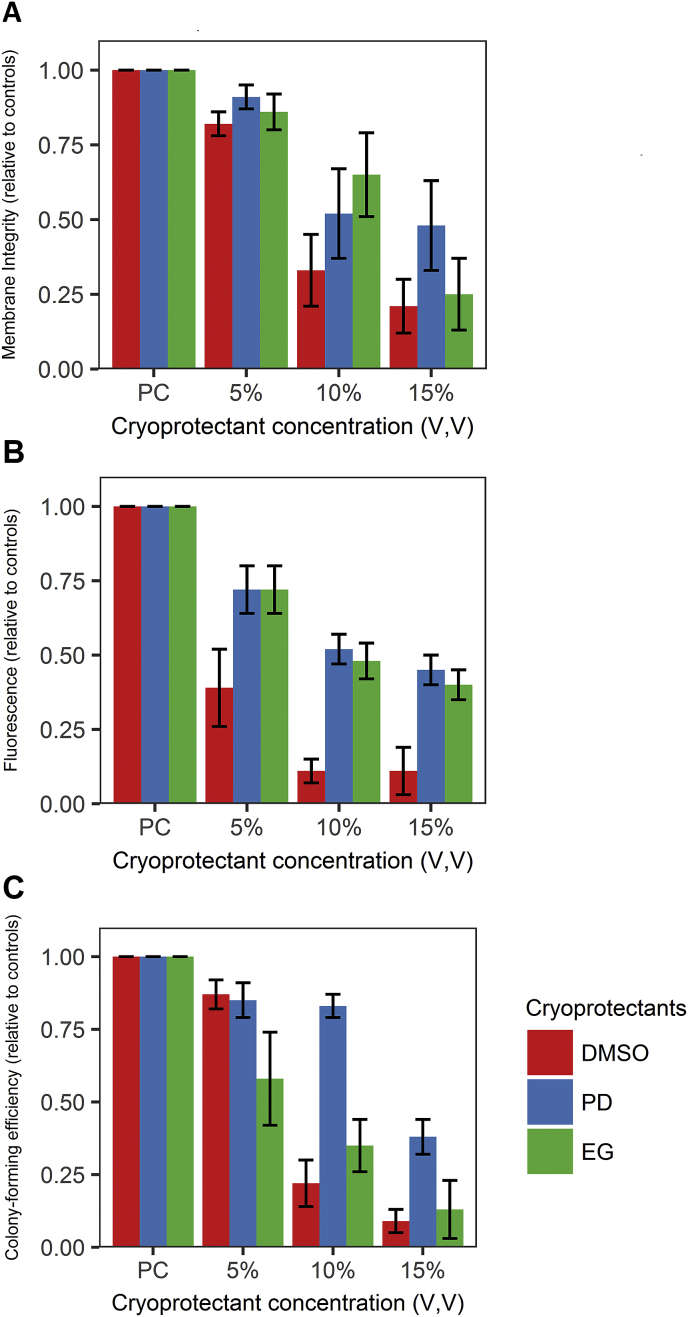
Bar graphs showing tolerance of limbal suspension cultures for each cryoprotectant at 5%, 10% and 15% concentrations, scaled to the positive controls. **(A)** mean membrane integrity ± SEM of limbal suspension cultures as measured by the Trypan blue assay after cooling to 4 °C for 10 min with either Me_2_SO, PD or EG at concentrations of either 5%, 10% or 15% by volume (n = 3). (**B)** mean metabolic activity ± SEM of limbal suspension cultures as measured by the alamarBlue^®^ assay after cooling to 4 °C for 10 min with either Me_2_SO, PD or EG at concentrations of either 5%, 10% or 15% by volume (n = 3). (**C)** mean colony-forming efficiency ±SEM of limbal suspension cultures after cooling to 4 °C for 10 min with either Me_2_SO, PD or EG at concentrations of either 5%, 10% or 15% by volume (n = 3). (For interpretation of the references to colour in this figure legend, the reader is referred to the Web version of this article.)

To analyse the impact of three different cryoprotectants and their concentrations we performed membrane integrity (MI), cellular metabolic assays (alamarBlue^®^ reduction) and colony-forming efficiency assays (CFE) (Fig. 2A–C). We found that increasing cryoprotectant concentration over 5% reduces membrane integrity, metabolic activity and colony-forming efficiency (p < 0.0001, p = 0.002, p < 0.0001, respectively), while choice of cryoprotectant has no significant effect (p = 0.9, p = 0.18, p = 0.25, respectively). The interaction between concentration and choice of cryoprotectant did not significantly account for changes in membrane integrity, metabolic activity or CFE (p = 0.70, p = 0.95, p = 0.29, respectively). Although choice of cryoprotectant did not account for a significant part of the variance in alamarBlue^®^ reduction, comparison between cryoprotectants by ANOVA showed Me_2_SO maintained metabolic activity significantly less than PD and EG (p = 0.008, p = 0.02, respectively; Fig. 2B).

**Fig. 3:**
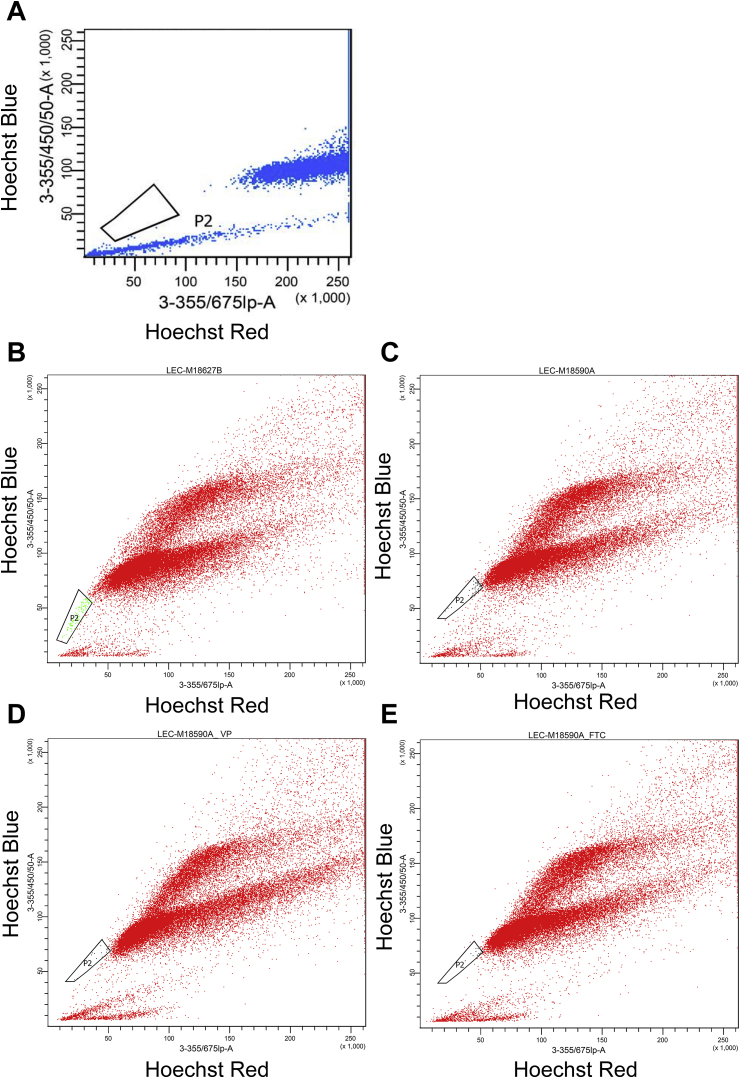
Flow cytometry plots of cryopreserved and thawed limbal suspension cultures showing the presence or absence of a Side Population fraction which extrude Hoechst. **(A)** uncryopreserved, cultured human fibroblasts in the presence of Hoechst. The trapezoid encapsulates the SP fraction (P2). **(B)** uncryopreserved human limbal epithelial cells in the presence of Hoechst. The trapezoid encapsulates the SP fraction (P2 – shown in green). **(C)** cryopreserved cultured human limbal epithelial cells in the presence of Hoechst. The trapezoid encapsulates the SP fraction (P2 – shown in green). The SP fraction in cryopreserved cultured human limbal epithelial cells is obliterated by incubation with Verapamil **(D)** and Fumitremorgin **(E)**. (For interpretation of the references to colour in this figure legend, the reader is referred to the Web version of this article.)

For the SP experiments, cultured limbal cell suspension samples were cryopreserved with 5% PD and frozen for 1 week, as described above. An SP fraction is not present in human fibroblasts (negative control) (Fig. 3A) and is present in uncryopreserved limbal epithelial cultures, with a sub-population ‘P2’ (green) effluxing the dye (Fig. 3B; P2 = 0.3%). The presence of an SP fraction in cryopreserved limbal suspension cultures is shown in Fig. 3C. P2 represented 0.1% of the total population analysed and 79.8% of cells had intact membranes. This P2 population is obliterated by incubation with Verapamil (a calcium channel blocker; Fig. 3D) and also with Fumitremorgin (a specific blocker of ABCG2; Fig. 3E). Therefore, cryopreservation for 1 week in the vapour phase of liquid nitrogen with 5% PD preserved a population of cells which exhibited Verapamil and Fumitremorgin-dependant efflux of Hoechst. Hoechst is known to be extruded by the ABCG2 channel, a human stem cell marker which is highly conserved across tissue types [4,16]. These results show that the P2 fraction represents cells exhibiting the stem cell phenotype which are likely to be limbal stem cells.

Increasing cryoprotectant concentrations above 5% is detrimental to cell tolerance in our experiments. Despite measures to reduce the effect of osmotic stress, it is likely to play a role, especially during removal of the cryoprotectant by dilution which is likely to cause a transient increase in cell volume. This is supported by our finding of decreasing membrane integrity with cryoprotectant concentration and lower levels of membrane integrity as compared to Oh et al. [9]. Thus, reducing the effect of osmotic stress will be important in future improvements to the cryopreservation of limbal cultures. The results of other studies which used higher concentrations of calf serum suggest this may be a remedy due to its penetrating cryoprotectant properties [9,15]. Despite all cryoprotectants showing similar levels of membrane integrity, cultures exposed to Me_2_SO showed reduced metabolic activity as compared to EG and PD. It is possible that Me_2_SO produces an artefactual reduction in alamarBlue^®^ reduction as it is known to inhibit components of the respiratory chain [3]. Finally, we have shown that cells exhibiting stem cell characteristics are preserved after cryopreservation with 5% PD, and therefore cryopreservation of limbal suspension cultures with PD has a potential future role in eye banking.

## Declarations of interest

None.
